# Natural IgM and TLR Agonists Switch Murine Splenic Pan-B to “Regulatory” Cells That Suppress Ischemia-Induced Innate Inflammation *via* Regulating NKT-1 Cells

**DOI:** 10.3389/fimmu.2017.00974

**Published:** 2017-08-23

**Authors:** Peter I. Lobo, Kailo H. Schlegel, Amandeep Bajwa, Liping Huang, Mark D. Okusa

**Affiliations:** ^1^Division of Nephrology, Center for Immunity, Inflammation and Regenerative Medicine, University of Virginia, Charlottesville, VA, United States

**Keywords:** natural IgM, IgM anti-leukocyte antibodies, Bregs, CpG, innate inflammation, ischemia–reperfusion injury, regulatory bone marrow dendritic cells, NKT-1

## Abstract

Natural IgM anti-leukocyte autoantibodies (IgM-ALAs) inhibit inflammation by several mechanisms. Here, we show that pan-B cells and bone marrow-derived dendritic cells (BMDCs) are switched to regulatory cells when pretreated *ex vivo* with IgM. B cells are also switched to regulatory cells when pretreated *ex vivo* with CpG but not with LPS. Pre-emptive infusion of such *ex vivo* induced regulatory cells protects C57BL/6 mice from ischemia-induced acute kidney injury (AKI) *via* regulation of *in vivo* NKT-1 cells, which normally amplify the innate inflammatory response to DAMPS released after reperfusion of the ischemic kidney. Such *ex vivo* induced regulatory pan-B cells and BMDC express low CD1d and inhibit inflammation by regulating *in vivo* NKT-1 in the context of low-lipid antigen presentation and by a mechanism that requires costimulatory molecules, CD1d, PDL1/PD1, and IL10. Second, LPS and CpG have opposite effects on induction of regulatory activity in BMDC and B cells. LPS enhances regulatory activity of IgM-pretreated BMDC but negates the IgM-induced regulatory activity in B cells, while CpG, with or without IgM pretreatment, induces regulatory activity in B cells but not in BMDC. Differences in the response of pan-B and dendritic cells to LPS and CpG, especially in the presence of IgM-ALA, may have relevance during infections and inflammatory disorders where there is an increased IgM-ALA and release of TLRs 4 and 9 ligands. *Ex vivo* induced regulatory pan-B cells could have therapeutic relevance as these easily available cells can be pre-emptively infused to prevent AKI that can occur during open heart surgery or in transplant recipients receiving deceased donor organs.

## Introduction

Several observations clearly demonstrate that both innate and adaptive immune inflammatory responses are suppressed by different regulatory mechanisms to protect the host from an over exuberant inflammatory response and from initiating autoimmune diseases. One such newly described regulatory mechanism is natural IgM, which are produced at birth by B1 cells, independently of exposure to foreign antigens and without the need for T helper cells [reviewed in Ref. (1, 2)]. Natural IgM are polyclonal autoantibodies with differing specificities, some of which have been identified, for example, IgM with specificity to leukocytes, erythrocytes, phosphorylcholine (PC) present on oxidized lipids, apoptotic cells, IgG (referred to as IgM rheumatoid factor), intracellular enzymes, DNA, and complement proteins. Several studies have shown that such autoantibodies are produced to prevent autoimmune-mediated inflammation [reviewed in Ref. (2)]. For example, IgM anti-PC, by binding to PC on oxidized lipids and apoptotic cell membranes, prevents immune activation by blocking neoantigen recognition and enhancing their removal by forming immune complexes that are phagocytosed by immature dendritic cell (DC) and macrophages.

Our laboratory has been interested in IgM anti-leukocyte autoantibodies (IgM-ALAs), which is a subset of natural IgM that binds to non-apoptotic leukocyte membrane receptors [reviewed in Ref. (1)]. Prior observations over the last 40 years, particularly in humans, have clearly demonstrated that IgM-ALA levels increase with various infections (viral and parasitic), autoimmune diseases (SLE), and other inflammatory states (sarcoidosis and end stage renal disease), and IgM-ALA levels normalize with control of infection or the inflammatory state [reviewed in Ref. (3)]. We hypothesized that natural IgM-ALA rapidly increases in <72 h to suppress an exuberant inflammatory response that could occur with infective and inflammatory states as they exhibit certain unique characteristics that are suited for such a purpose. First, IgM-ALAs have been shown to bind to specific leukocyte receptors involved in initiating the inflammatory response (e.g., CD3, CD4, CD40, CD86, and PD1) and in leukocyte trafficking (CXCR4 and CCR5) (3–5). Second, unlike pathogenic IgG autoantibodies, IgM-ALAs have low binding affinity to cells, and at body temperature, the cell-bound IgM does not activate the lytic components of complement. Hence, at body temperature, they are particularly suited to modulate cell receptors without inducing complement-mediated cell damage (6–8). Third, these large pentameric autoantibodies predominantly reside in the vascular compartment and not only bind to leukocytes but also bind to receptors on endothelial cells and inhibit activation of endothelial cells (9).

In support of a regulatory role for IgM-ALA are observations in patients with high levels of IgM-ALA. Such patients have significantly less rejections and prolonged kidney and heart survival after a transplant (10–14), and second, these patients develop significantly lower titers of anti-HLA antibodies after alloantigen sensitization while waiting for a transplant (8). In subsequent studies with murine models of inflammation, we showed that administration of intravenous polyclonal IgM, to increase plasma IgM-ALA levels in wild-type C57BL6 mice (referred to as WT-B6), attenuated the inflammatory response induced by innate (renal ischemia), adaptive (cardiac transplants), and autoimmune (diabetes mellitus in NOD mice)-mediated mechanisms (9, 15). Specifically, we showed that protection was mediated by the IgM-ALA subset present in the purified polyclonal IgM preparation as the protective effect on ischemia-induced acute kidney injury (AKI) was abolished when polyclonal IgM was depleted of IgM-ALA with leukocyte adsorption (9).

The several fold higher binding of IgM-ALA to splenic CD11c^+^ DC when compared to T cells prompted us to investigate whether IgM-ALA inhibited the innate inflammatory response in part, by modulating the function of DC during antigen presentation. In these studies, bone marrow-derived dendritic cells (BMDC) were cultured *ex vivo* for 48 h with IgM, and such IgM-pretreated BMDC, when pre-emptively infused, protected WT-B6 mice from ischemia-induced AKI by inhibiting the innate inflammatory response that occurs after reperfusion (4). Such protection was not observed with pre-emptive infusion of IgM-pretreated T cells. We showed that IgM pretreatment switched BMDC to a regulatory phenotype, which required PD1 and IL10 to inhibit the inflammatory response. Furthermore, we showed that LPS enhanced the regulatory activity of IgM-pretreated BMDC.

The current studies were aimed at determining the functional impact of IgM-ALA on splenic pan-B cells especially since we also observed threefold to fourfold more IgM binding to B cells when compared to splenic DC (4). As with BMDC, we show that *ex vivo* pretreatment of splenic pan-B cells with IgM also induced regulatory activity in B cells, which when intravenously infused, protected mice from subsequent ischemia-induced AKI. In addition, we show that CpG, but not LPS, enhanced the IgM-induced regulatory activity of B cells. We also show that *ex vivo* induced regulatory B cells and BMDC inhibit the inflammatory response *in vivo* by regulating NKT-1 cells, which normally amplify the renal ischemia-induced innate inflammatory response in WT-B6 mice (16, 17).

## Materials and Methods

### IgM and IgG Purification from Plasma

IgM and IgG were purified from heat-inactivated (56°C) WT-B6 murine plasma or normal human plasma using size exclusion column chromatography (Sephacryl S-300 HR) as previously described (4) except for modifications detailed below. Care was taken to separate plasma within 4 h of obtaining blood, and then plasma was sterile filtered to remove any contaminating bacteria. Plasma was stored at 4°C prior to column purification. IgM was not isolated by dialyzing sera in water or by ammonium chloride precipitation as both these techniques yielded IgM with impaired functional activity. Column purified IgM was re-passaged through Sephacryl S-300 to remove contaminating proteins that were not effectively removed with the first passage. Residual IgG in the IgM preparation was removed by using agarose beads covalently linked to goat anti-murine IgG. Protein A was not used as any leftover protein A in the IgM preparation also altered lymphocyte function. Purified IgM and IgG was concentrated to 1.3–1.5 mg/ml and then dialyzed against RPMI-1640 and sterile filtered prior to use in cultures and for *in vivo* use. Purified IgM was stored at 4°C and not frozen as functional activity of IgM was reduced with freezing. The effect of IgM on *in vitro* cultures was dose dependent, and the maximum effect of IgM was observed using IgM at a physiological dose of 10–30 μg/250 × 10^3^ cells/0.5 ml of culture. We used two control IgM, i.e., our purified mouse IgM (polyclonal) adsorbed with WT-B6 splenic leukocytes to remove IgM-ALA (see Ref. (4) for adsorption details) and a monoclonal mouse IgM anti-KLH (clone C48-6) obtained from BD Pharmingen.

Permission was obtained from our institutional review board to obtain blood from normal human volunteers for this study.

### Mice

We obtained C57BL6 mice (WT-B6) from Charles River Laboratories. IL10 ko (B6.129P2-IL10*^tm1Cgn^*/J), CD80/86 ko (B6.129S4-*Cd80^tm1Shr^Cd86^tm2Shr^*/J), CD40 ko (B6.129P2-CD40*^tm1Kik^*/J), and Rag-1 ko (B6.129S7-*Rag1^tm1Mom^*/J) on a C57/BL6 background were from Jackson Laboratories.

### Surgical Protocol for Kidney Ischemia–Reperfusion Injury (IRI)

All experiments were performed in accordance with NIH and Institutional Animal Care and Use Guidelines. The Animal Research Committee of the University of Virginia approved all procedures and protocols. All experiments were performed on 8- to 10-week-old male mice, weighing approximately 20 g. Kidney IRI was performed under anesthesia with bilateral flank incisions as we have previously described (4). Both kidney pedicles, consisting of renal artery and vein, were exposed and cross-clamped for 26 min, and then clamps were released and kidneys were re-perfused for 24 h. Body temperature was maintained at 34.5–35°C (rectal temp) during surgery with heating pads. Kidney pedicles were exposed, but not clamped in sham-operated mice. Postoperatively, mice were administered analgesia and maintained at 35–37°C. For splenectomy, mice were anesthetized and *via* a flank incision, the spleen was exposed, and after ligation of splenic vessels, the spleen was removed. Splenectomy was performed 7 days before mice were subjected to renal IRI.

### Assessment of Kidney Function and Histology after Renal Ischemia (IRI)

Plasma creatinine was determined 24 h after renal IRI using an enzymatic assay (Diazyme Laboratories, Poway, CA, USA) according to the manufacturer’s protocol. Kidney histology and quantitation of tubular injury were assessed at 24 h using previously described techniques (4).

### Isolation of Splenic Pan-B Cells and BMDC and *Ex Vivo* Pretreatment of These Cells

Pan-B cells, B1a cells, and follicular (FO) B cells were obtained from murine spleens using magnetic microbead isolation columns specific for Pan-B, B1a, and MZ/FO (Miltenyi Biotech Inc., San Diego, CA, USA) and following instructions in the kit. B1a (CD5^+^) and FO (CD23^+^) B cell subsets were isolated in a second step using positive selection. Pan-B cells consisting of B1 and B2 cells were obtained by negative selection. Isolated B cell subsets were >92% pure.

BMDCs were generated from bone marrow that was obtained from murine femurs and cultured in RPMI containing 10% FCS and 2 ng/ml of recombinant GM-CSF as previously described (4). Media was changed every 3 days and supplemented with GM-CSF. Non-adherent cells were used, and after 8–10 days, >90% of these non-adherent cells expressed moderate to high levels of CD11c/MHC-Class II and <15% were GR1 positive.

*Ex vivo* pretreatment of B cells and BMDC (7–10 days old) involved culturing 1 × 10^6^ cells in 1 ml media in 5 ml falcon plastic tubes for 36–48 h in the presence of LPS (*Escherichia coli*, 400 ng/ml), CpG (ODN 2395 class C. InvivoGen, 5 μg/ml), and polyclonal IgM (100 μg/ml). IgM was added 40–60 min before LPS or CpG. For controls, we used non-CpG ODN and isotype murine IgM [i.e., IgM anti-hemocyanin, IgM anti-KLH (clone C48-6), BD Pharmingen]. In certain experiments, B cells were activated for 48 h with an agonistic anti-CD40 monoclonal antibody (clone ICD10, Jackson Immunoresearch, West Grove, PA, USA, 1 μg/ml), which was cross-linked by goat anti-rat IgG (Affymetrix). After the culture period, cells were washed two times to remove unbound LPS and IgM, and 0.5 × 10^6^ pretreated BMDC or B cells were injected in the tail vein of each mouse. In certain experiments, anti-CD86 (clone 1D10), PD1 (clone J43), PDL1 (clone MIH5), and CD1d (clone 1D1) blocking antibodies (5 μg/ml) or their isotype controls (obtained from eBioscience) were added to cells after the 48-h culture to block these receptors prior to infusing into mice. Viability of 48 h pretreated B cells and BMDC was >70% as assessed by live/dead stain with flow cytometry.

### Induction of Apoptosis in B Cells

Splenic pan-B cells pretreated with IgM for 48 h at 37°C were exposed to 150 mJ/cm^2^ of ultraviolet C irradiation (Stratagene Stratalinker 2400, Fisher Scientific). Irradiated cells were incubated for 6 h at 37°C to allow for IgM binding to apoptotic cells. Cells were washed prior to intravenous injection into mice. More than 80% of cells were apoptotic (Annexin V+) at the time of injection.

### Infusion of Mice with Splenic Pan-B Cells or BMDC

In these experiments, *ex vivo* pretreated isolated pan-B cells or BMDC (0.5 × 10^6^ cells in 0.5 ml) were infused *via* the tail vein. Bilateral renal ischemia (26 min) was performed 24 h after the infusion. Mice were sacrificed 20–24 h after renal reperfusion.

### Antibodies and Other Reagents

The following monoclonal antibodies were used for flow cytometry or immunostaining of tissues and were obtained from eBioscience, San Diego, CA, USA: anti-PD1 (clone J43), anti-PDL1 (clone MIH5), anti-CD40 (clone 1C10), anti-CD80 (clone 16-10A1), anti-CD86 (clone GL1), anti-CD11c (clone N418), anti-CD3 (clone 145-2C11), anti-CD19 (clone eBio 1D3), and anti-IgM (clone II/41). Appropriate fluorochrome-conjugated, isotype-matched irrelevant mAbs were also obtained from eBioscience. Other reagents LPS-*E. coli* 0111:B4 (Sigma-Aldrich, St. Louis, MO, USA) and Live/Dead fixable Kit were used (Life Technology, Grand Island, NY). TUNEL *in situ* cell death detection kit was obtained from Roche diagnostics (IN, USA).

### Immunofluoresence Staining of Splenic Pan-B Cells and BMDC

9- to 12-day BMDCs were used in these studies. IgM-mediated receptor downregulation was evaluated by adding IgM 40–60 min before LPS at the initiation of the 37°C culture, which was terminated after 48 h. After culture termination, cells were washed and blocked with unlabeled anti-mouse CD16/32 (clone 2.4G2) prior to adding labeled antireceptor antibody. Appropriate fluorochrome-conjugated, isotype-matched, irrelevant mAbs were used as negative controls. Intracytoplasmic staining was performed using methods as described for splenic leukocytes.

### Immunofluoresence Staining of Kidney Sections

Kidney tissue was fixed and frozen as we have previously described in detail (4). Frozen sections (5 μm) of kidney were permeabilized with 0.3% Triton X-100, and non-specific binding was blocked with 10% horse serum and anti-mouse CD16/32. Tissue sections were stained with the following conjugated antibodies: rat anti-Ly-6B.2 (anti-neutrophil, clone 7/4, Bio-Rad, Raleigh, NC, USA) and rat anti CD31 (anti-endothelial cell, clone 390, eBioscience). Nuclei were visualized using DAPI.

### Quantitation of Cytokines in 48 h Supernatants of *Ex Vivo* Induced Regulatory B Cells and BMDC

Cytokines were quantitated on supernatants that were immediately frozen at −80°C. Our flow cytometry core Lab at University of Virginia performed these studies using a Luminex bead assay (obtained from Millipore/Sigma, Billerica, MA, USA) that quantitated a panel of 32 proteins consisting of cytokines (including IFNγ, IL1α, IL1β, IL2, IL4, IL3, IL5, IL6, IL7, IL9, L10, IL12p40, IL12p70, LIF, IL13, IL15, IL17, TNFα, and LIX) as well as chemokines (including IP10, KC, MCP-1, MIP-1α, MIP-1β, M-CSF, MIP-2, MIG, and RANTES) and a separate panel of Luminex beads that only quantitated active (but not pro) TGFβ1, TGFβ2, and TGFβ3. B cells produced no or very low levels (<5 pg/ml) of IL1α, IL1β, IL2, IL4, IL3, IL5, IL7, IL9, IL-12, LIF, IL13, LIX, IL15, IL17, TGFβ2, and TGFβ3 even when activated with CpG or LPS. Except for M-CSF, B cells produced all the tested chemokines that significantly increased with TLR activation.

### Statistics

Data in figures were analyzed by one-way ANOVA except where indicated. *P* < 0.05 was used to indicate significance.

## Results

### IgM and TLR Ligands Utilize Different Mechanisms to Regulate Pan-B Cells and BMDC

**Figure 1:**
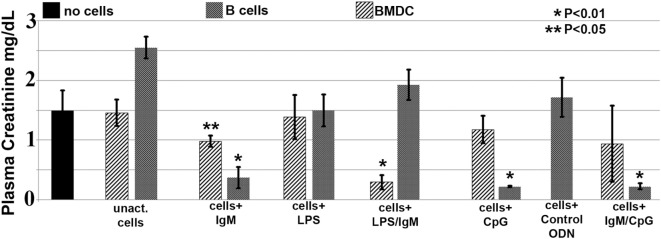
IgM-pretreated B cells are more protective than IgM-pretreated BMDC in preventing ischemia-induced acute kidney injury. Unlike with BMDC, addition of LPS negates the protective effect of IgM-pretreated B cells. CpG activation, unlike LPS, renders B cells, but not BMDC, protective. Use of B cells pretreated with isotype control IgM was not protective (data not shown). Each data point is a mean of at least three separate experiments with at least six mice. * and ** denotes significance (one-way ANOVA) compared to renal ischemia without cell therapy (black bar).

**Figure 2:**
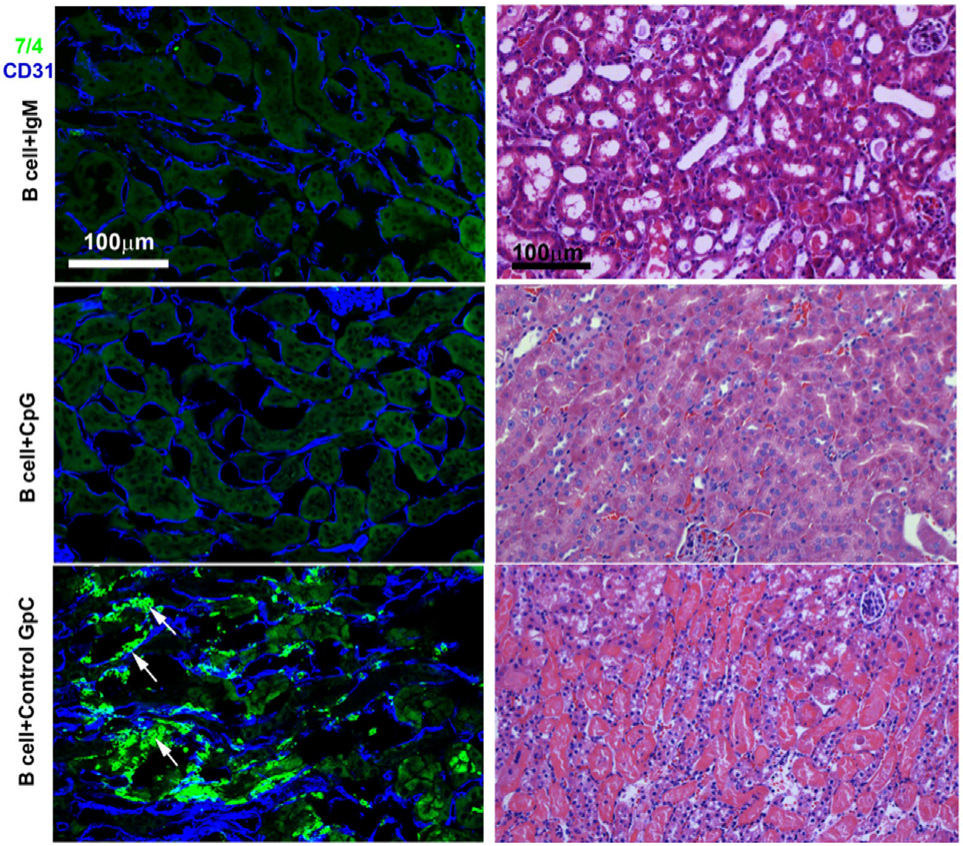
IgM- or CpG ODN-pretreated B cells protect against ischemia-induced renal injury by inhibiting the ischemia-induced inflammatory response (upper two panels). On immunofluorescence staining, note no or minimal 7/4+ inflammatory cell (bright green) infiltration in protected kidneys. CD31^+^ endothelial cells (blue) are well preserved in protected kidneys. Infusion of B cells pretreated with control GpC ODN failed to protect (bottom panel). Note significant infiltration with 7/4+ inflammatory cells (bright green) many having bilobed nuclei resembling neutrophils (arrows) and damaged CD31^+^ endothelial cells (blue). The Ly-6B.2 antigen detected by clone 7/4 is expressed on all neutrophils and activated monocytes/macrophages. On H&E-stained histology, note preservation of outer medulla renal tubular histology with minimal interstitial inflammation when mice were infused with IgM- or CpG ODN-pretreated B cells before renal ischemia. There are loss of tubular cells, severe inflammation, and capillary damage with interstitial hemorrhage when mice were infused with B cells pretreated with control GpC ODN. This is a representative example from four separate experiments.

In these studies, we wanted to evaluate if *ex vivo* pretreatment with IgM switches B cells to a regulatory phenotype especially since, in prior studies, we observed that IgM has several fold higher binding to B cells when compared to splenic DCs (4). Column purified splenic pan-B cells were pretreated with IgM *ex vivo*, and 0.5 × 10^6^ pretreated pan-B cells were intravenously infused into mice 24 h prior to performing renal ischemia. Such *ex vivo* IgM-pretreated pan-B cells effectively protected mice from ischemic AKI and adding LPS during the IgM pretreatment negated the regulatory effect of IgM on B cells (Figure 1). As in prior studies with BMDC (4), infusion of pan-B cells, pretreated *ex vivo* with leukocyte adsorbed IgM (control IgM), failed to protect mice from ischemic AKI (data not shown). The opposite effect of LPS on B cells vs BMDC prompted us to evaluate whether other TLRs were involved in the induction of regulatory activity in B cells and BMDC (Figure 1). We therefore tested our hypothesis by using another TLR agonist, i.e., Class C CpG, a TLR9 agonist. CpG, in the absence of IgM, induced regulatory activity in B cells, but not in BMDC, and CpG enhanced the regulatory effect of IgM on pan-B cells, and unlike LPS, CpG negated the protective effect of IgM on BMDC (Figure 1). As depicted in Figure 2, protected kidneys had relatively well-preserved tubular and endothelial cells as well as no or minimal granulocyte infiltration, thus indicating that the infused *ex vivo* pretreated B cells inhibited the ischemia-induced inflammatory response that causes renal tubular and endothelial cell injury.

### IgM Induces Regulatory Activity in B1a and FO B2 Cells through a Mechanism Not Involving B Cell Apoptosis

**Figure 3:**
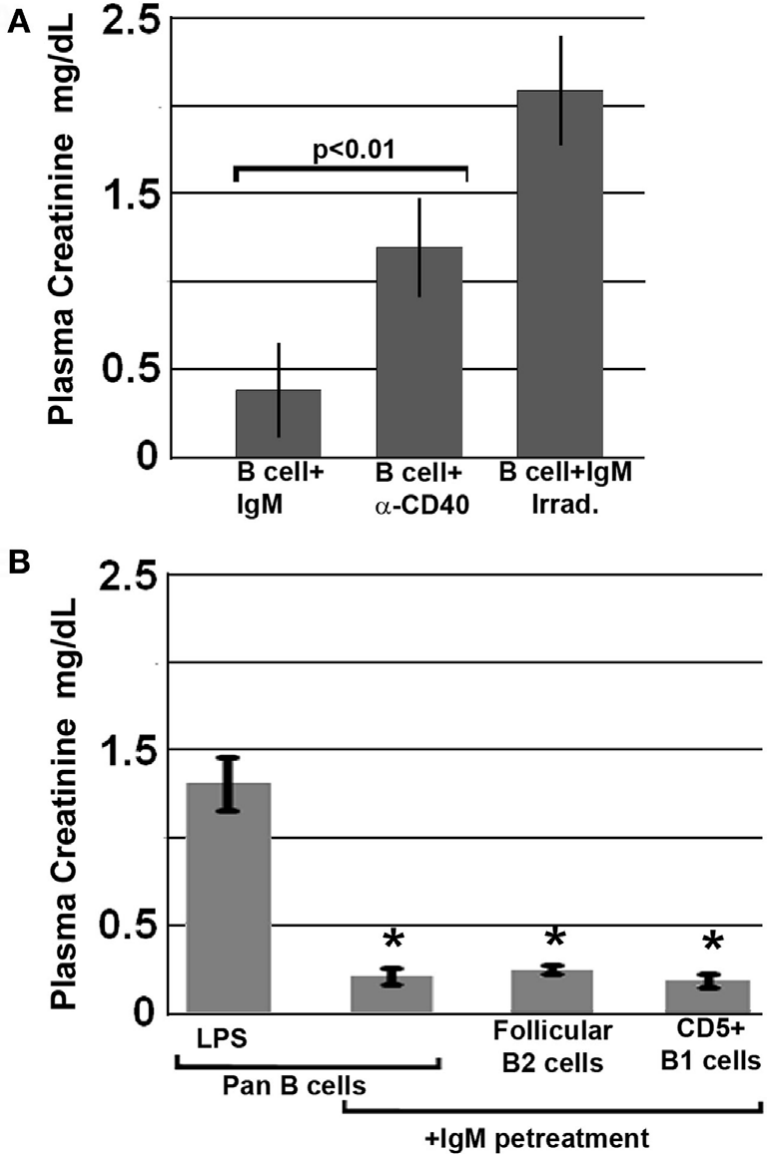
All splenic B cell subsets pretreated *ex vivo* with IgM are switched to a regulatory phenotype. Bregs lose regulatory activity when irradiated or activated with anti-CD40. **(A)** IgM-pretreated B cells fail to protect when irradiated just before infusion. Similarly, B cells pretreated with an agonistic anti-CD40 antibody that was also cross-linked are non-protective. **(B)** IgM-pretreated Pan-B and B cell subsets are equally protective. Data are a mean of three separate experiments with each experimental group having three mice. *Significance when compared to LPS-pretreated pan-B cells.

Further studies were aimed at defining how IgM induces regulatory activity in pan-B cells. Since others have shown that infusion of apoptotic cells can induce *in vivo* Bregs (18), and since up to 30% of the infused *ex vivo* pretreated B cells were apoptotic, we examined this possibility by infusing B cells that were rendered >80% apoptotic with UV irradiation, i.e., after they were pretreated for 48 h with IgM. Data in Figure 3A show that apoptotic B cells, induced by UV irradiation, failed to protect mice from renal ischemia-induced AKI, thus indicating that protection from ischemia-induced AKI was mediated by the live subset of infused IgM-pretreated B cells, which regulate the ischemia-induced inflammatory response. Similarly, *ex vivo* induced regulatory BMDC failed to be protective in ischemia-induced renal injury when they were rendered apoptotic (4). Hence, these studies would indicate that IgM induces regulatory activity in pan-B cells and BMDC by possibly binding to some undefined inhibitory receptor expressed by these cells.

We next determined whether *ex vivo* pretreatment of pan-B cells with IgM-induced regulatory activity in B cell subsets arising from certain splenic B cell compartments, i.e., follicles or marginal zone. This question was evaluated by using the Miltenyi Biotec Kit and their MACS columns to isolate pan-B cells, FO B2 cells, or CD5 + B1a cells from murine spleens. Data in Figure 3B show that infusion of 0.5 × 10^6^ pan-B cells, FO B2, or B1a cells, pretreated *ex vivo* with IgM, protected mice from ischemia-induced renal injury indicating; therefore, that IgM induces regulatory activity in Breg subsets present in the splenic B1a and FO B2 cell compartments.

### Human IgM-Pretreated Human B Cells Protect Mice after Renal Ischemia

**Figure 4:**
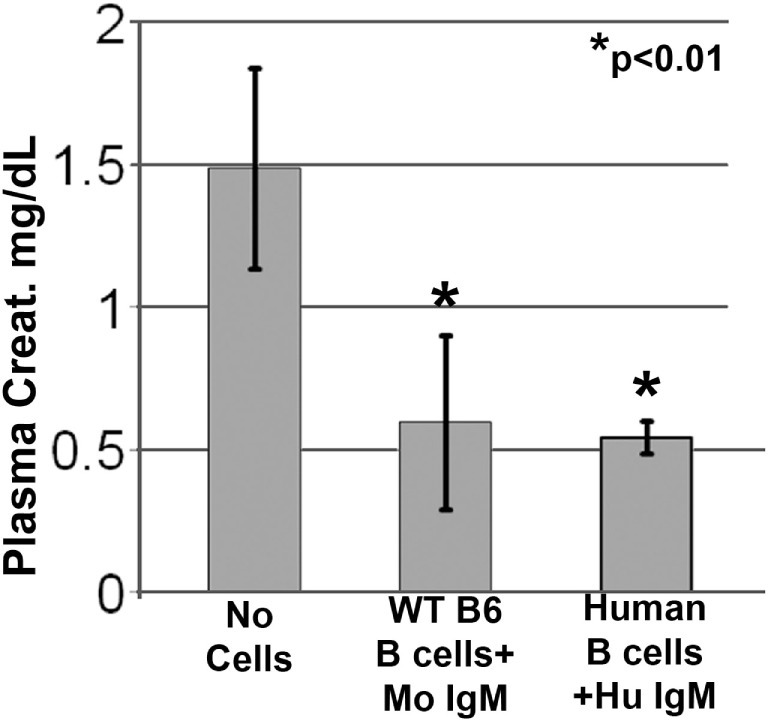
Human IgM pretreatment of human B cells obtained from human blood and murine IgM pretreatment of murine B cells obtained from WT-B6 spleens are protective in renal ischemia. *p value compared to No Cells (one-way ANOVA). Data are a mean of three separate experiments.

Since natural IgM is evolutionarily conserved and human IgM can bind to both murine and human B cells, we examined if natural human IgM can regulate human B cells (1, 19). Such human IgM-pretreated human B cells were tested in the murine model of renal ischemia, as in this model, the infused cells exert their functional effects in <48 h, i.e., before these cells are rejected. As shown in Figure 4, infusion of 0.5 × 10^6^ human IgM-pretreated human B cells protected mice from renal ischemia-induced AKI. Therefore, these studies indicate that human IgM and B cells can inhibit the ischemia-induced innate inflammatory response in mice by a mechanism that is also evolutionarily conserved.

### *Ex Vivo* Induced Regulatory B Cells and BMDC Inhibit Renal Ischemia Induced Innate Inflammation by Regulating *In Vivo* NKT-1 Cells

**Figure 5:**
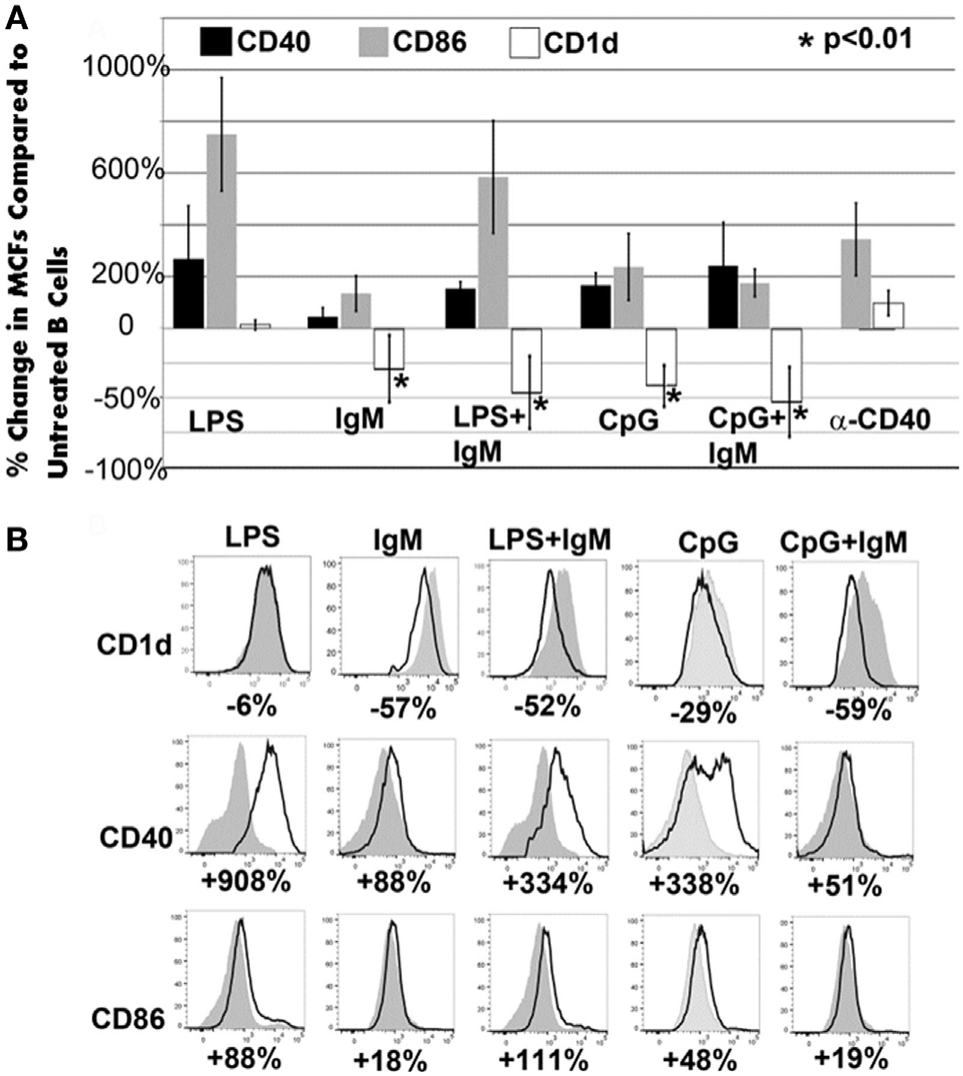
CD1d is downregulated with all *ex vivo* pretreatments that induce regulatory activity in pan-B and BMDC. **(A)** Pretreatment of pan-B cells: in all protective pretreatments (i.e., IgM or CpG or CpG/IgM), B cells express decreased CD1d when compared to unactivated cells. Of the three non-protective pretreatments (LPS or LPS + IgM or anti-CD40), only LPS + IgM-pretreated B cells exhibited CD1d downregulation. There is an increased CD86 and CD40 expression when compared to non-activated pan-B cells in both protective and non-protective B cells. Unactivated and pretreated cells were cultured for 48 h with IgM, CpG, or LPS. Change in receptor expression over unactivated cells is expressed as % change. Downregulation is depicted as negative (−) percent change. Asterisks denote significance when the % change is compared to CD1d expression on LPS-pretreated cells (one-way ANOVA). **(B)** Pretreatment of BMDC: representative example from one of four different experiments is depicted. Note that all protective (IgM or IgM + LPS) BMDCs have significant downregulation of CD1d. However, some non-protective BMDC (i.e., CpG or CpG + IgM) also exhibited downregulation of CD1d. Gray histogram represent unactivated cells. % represents change in receptor expression over unactivated cells. Each data point is a mean of three separate experiments with each experiment having three mice per group.

Acute renal ischemia releases DAMPS and lipid antigen, which normally activate NKT-1 to release large amounts of IFNγ that amplifies the innate inflammatory response (16, 17). We therefore questioned whether infused regulatory B cells or BMDC inhibited *in vivo* NKT-1 cell function especially since CD1d expression on these regulatory cells was significantly downregulated after *ex vivo* pretreatment with IgM (Figure 5). Such a possibility seemed plausible as intravenously infused cells have been shown to rapidly enter the splenic marginal zone as well as the liver where they can interact with *in vivo* NKT-1 cells (20, 21), and second NKT-1 activation by lipid antigen in the context of low CD1d expression has been shown to decrease NKT cytotoxicity and INFγ production (20, 22). Since there was downregulation of CD1d in all *ex vivo* pretreatment conditions that generated regulatory pan-B or BMDC, we reasoned that infused cells expressing low CD1d could regulate *in vivo* NKT-1 cells and such NKT-1 cells with a regulatory phenotype may not be able to produce high levels of IFNγ when re-challenged by lipid antigen released after ischemia.

To test the hypothesis that *ex vivo* pretreated regulatory B cells and BMDC regulate *in vivo* NKT-1 cells, we employed four approaches. We initially evaluated if NKT-1 cells had an important role in mediating inflammation in our model of renal ischemia. In this approach, we used WT-B6 mice where NKT-1 cells were rendered anergic. Second, we evaluated if the *ex vivo* induced regulatory cells required CD1d and costimulatory receptors to interact *in vivo* with NKT-1 cells to inhibit the ischemia-induced innate inflammatory response. Third, we examined if regulatory B cells, induced *ex vivo* by either IgM or CpG, require PD1/PDL1 or IL10 to mediate their *in vivo* regulatory effect on NKT-1 cells. Finally, we evaluated if infused regulatory BMDC and B cells regulated *in vivo* NKT-1 cells or induced NKT-1 cells to switch to a non-functional or anergic state.

#### Role of *In Vivo* NKT-1 in Ischemic AKI

**Figure 6:**
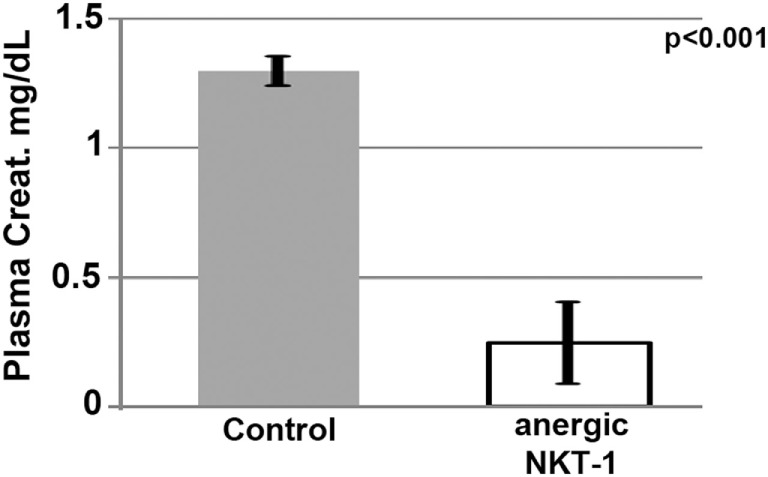
Lack of renal acute kidney injury in mice with anergic NKT-1 cells induced by intraperitoneal α-Gal-Cer (5 μg). Four mice were used in each group.

The role of *in vivo* NKT-1 cells in mediating the innate inflammatory response in our renal ischemia model was tested by performing renal ischemia 72 h after *in vivo* NKT-1 cells were rendered non-functional or anergic with a large bolus (5 μg) of intraperitoneal α-gal-ceramide as previously described (23). Data in Figure 6 show that 26 min of renal ischemia failed to induce AKI, as determined by an increase in serum creatinine, when *in vivo* NKT cells were rendered non-functional or anergic with a large bolus of α-gal-ceramide, thus indicating that NKT-1 cells have an important role in mediating the renal ischemia-induced inflammatory response that leads to renal injury in our model of ischemia. In prior studies, using other approaches, including Jα18ko mice, we have also shown that NKT-1 cells by producing high levels of IFNγ have an important role in amplifying the innate inflammatory response in our renal ischemia model (16).

#### Interaction of Infused Regulatory Cells with *In Vivo* NKT-1

**Figure 7:**
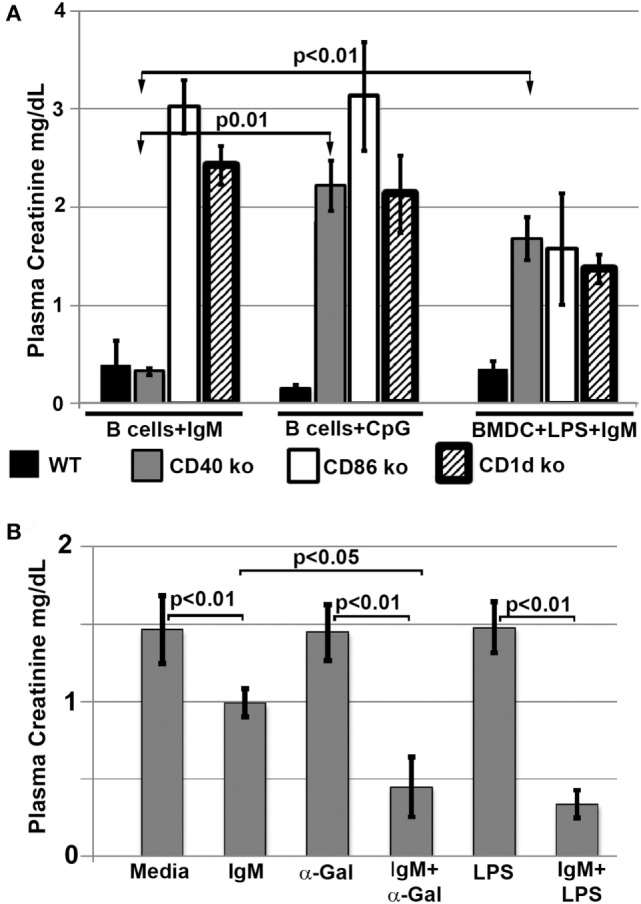
**(A)** Regulatory B cells and BMDC require CD86, CD40, and CD1d to interact with *in vivo* NKT. Note that IgM-pretreated regulatory B cells do not need CD40 to interact with NKT-1 cells. **(B)** α-Gal-Cer or LPS enhance protection mediated by IgM-pretreated BMDC on renal ischemia. BMDCs were cultured with LPS (400 ng/ml) or α-Gal-Cer (100 ng/ml) in the presence of IgM for 48 h at 37°C, washed, and then infused into mice. Data are a mean of at least three separate experiments, and three mice were used for each group in each experiment.

**Figure 8:**
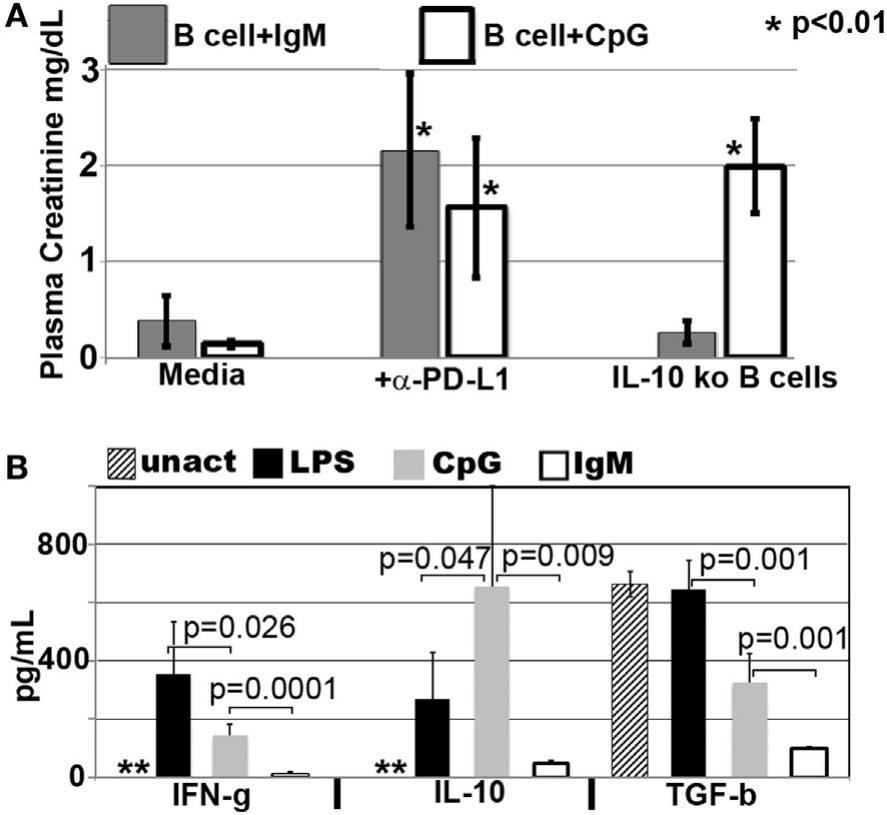
CpG-induced Bregs require PDL1 and IL10 to inhibit inflammation, while IgM-pretreated Bregs require PDL1 but not IL10 or TGFβ1. **(A)** IgM- and CpG-pretreated B cells lacked protection if PDL1 was blocked just prior to infusion. However, CpG, but not IgM-induced regulatory B cells, required IL10 to mediate *in vivo* protection. In blocking experiments, pretreated B cells were cultured for 48 h, washed, incubated with anti-PDL1 (5 μg/ml) or isotype control at 4°C for 1 h, re-washed, and then infused (IV) into mice. Isotype control α-PDL1 did not reverse the protective effect of regulatory B cells induced by IgM or CpG (data not shown). *p value compared to media. **(B)** Levels of IFNγ, IL10, and TGFβ1 present in 48-h culture supernatants of purified B cells that were not pretreated (labeled unact) or pretreated with LPS (400 ng/ml), CpG (5 μg/ml), or IgM (100 μg/ml) during the 48-h culture. 1 × 10^6^ B cells/ml were cultured for 48 h with purified IgM, CpG, or LPS or no additive. Supernatants were obtained before B cells were washed and tested for regulatory activity by intravenous infusion into mice that subsequently underwent renal ischemia. Data are obtained from six to eight different supernatants. **Untreated B cells secreted <1 pg/ml of cytokine. In methods, we provide profile of other cytokines that were also quantitated by the Luminex beads.

Since interaction of infused regulatory cells with *in vivo* NKT-1 cells requires CD1d to present glycolipids and appropriate costimulatory interaction, we infused *ex vivo* pretreated B cells and BMDC that were obtained from CD1d and CD 80/86 ko mice. Data in Figure 7A demonstrate that the infused regulatory B cells or IgM + LPS-pretreated regulatory BMDC needed both CD1d and CD80/86 to interact with *in vivo* NKT-1 cells and protect mice from ischemic AKI (16, 17). Data in Figure 7A also show that CpG but not IgM required CD40 to induce regulatory activity in B cells. This latter finding may be best explained on the role of CD40 in enhancing production of IL10 (24, 25), which is required by CpG-, but not IgM-, induced Bregs (data to be presented in Section “Pan-B Cells and BMDC Regulate *In Vivo* NKT-1 by Distinct Mechanisms”; Figure 8B).

In a second, more direct approach, we infused BMDC loaded *ex vivo* with α-gal-ceramide to directly interact with *in vivo* NKT-1 cells. As can be seen in Figure 7B, infusion of IgM-pretreated α-gal-ceramide loaded BMDC, expressing low CD1d (Figure 5B), protected mice from ischemic AKI. These studies indicate that protection was mediated by a direct interaction of infused IgM-induced regulatory BMDC (presenting low amounts of α-gal-cer) with *in vivo* NKT-1. Infusing IgM-pretreated BMDC (i.e., without α-gal-cer loading) was not as effective in protecting against ischemic AKI even though IgM downregulated CD1d (Figures 7B and 5B). However, infusing BMDC pretreated with both LPS and IgM was most effective in protecting against ischemic AKI (Figure 7B) possibly because LPS potentiated the inhibitory effect of IgM on BMDC by upregulating CD40 (Figure 5B), which has an important role in enhancing IL10 production after TLR activation (24, 25).

#### Pan-B Cells and BMDC Regulate *In Vivo* NKT-1 by Distinct Mechanisms

In prior studies, we showed that LPS + IgM-pretreated BMDC required PD1 (but not PDL1) and IL10 to regulate the *in vivo* innate inflammatory response mediated by renal ischemia (4). Therefore, we examined if regulatory B cells, induced *ex vivo* by either IgM or CpG, require PD1/PDL1 and IL10 to mediate their regulatory or suppressive effect on *in vivo* NKT-1 cells. This question was examined by using three approaches. First, *ex vivo* induced regulatory B cells were blocked with either anti-PD1 or anti-PDL1 just before *in vivo* cell infusion to determine if PD1/PDL1 were required. In these studies, *ex vivo* induced regulatory B cells were found to require PDL1 (Figure 8A), but not PD1 (data not shown), for their *in vivo* suppressive effect. Anti-PDL1 was more effective than anti-PD1 in blocking the *in vivo* inhibitory function of CpG- or IgM-induced regulatory B cells as these pretreated cells expressed high PDL1 and very low PD1 levels. B cells constitutively express high PDL1 and low PD1, and we, like others (26), failed to enhance PD1 expression even after LPS activation (data not shown).

Second, we used IL10 knock-out B cells to test if *ex vivo* induced regulatory B cells required IL10 to mediate their *in vivo* inhibitory effect. Rather surprising, IgM-pretreated B cells did not require IL10 to mediate their *in vivo* regulatory activity while CpG-pretreated B cells required IL10 (Figure 8A). Supernatants from IgM-pretreated B cells had in addition significantly decreased levels of TGFβ1 even when compared to untreated and CpG pretreated B cells (Figure 8B), thus indicating that IgM-pretreated B cells mediate regulatory activity by a mechanism that does not involve TGFβ1 or IL10. Currently, we have not delineated how IgM inhibits IFNγ, IL10, and TGFβ production. However, in the presence of low IFNγ production, B cells pretreated with IgM do not appear to require anti-inflammatory cytokines but require low Cd1d expression and PDL1 to regulate *in vivo* NKT-1. Such a hypothesis would also explain (i) the lack of regulatory activity in LPS-pretreated B cells where CD1d is not downregulated and production of IFNγ is significantly high when compared to IL10 (Figures 5A and 8B) and (ii) the presence of regulatory activity in CpG-pretreated B cells where CD1d is downregulated and the production of IL10 is significantly higher when compared to IFNγ production (Figures 5A and 8B). The high IL10 requirement for CpG-induced Bregs would also explain why CpG-, but not IgM-, induced Bregs are dependent on CD40 (Figure 7A), which when ligated by CD40L expressed by adjacent B cells in *ex vivo* culture or by NKT-1 *in vivo* enhances IL10 production of Bregs (24, 25).

Pretreating B cells with IgM or CpG or LPS did not change the 48-h supernatant levels of IL17, IL4, and IL13, which were produced at very low levels (<5 pg/ml) or TNFα, which was produced at low levels (30–110 pg/ml) (data not shown). Non-pretreated B cells produced no or very low levels (<5 pg/ml) of all cytokines except for high levels of TGFβ1 (Figure 8B). Interestingly, lack of IFNγ and production of high TGFβ1 by untreated B cells was not sufficient to induce regulatory activity in these cells (Figure 1), thus clearly indicating that regulatory activity in B cells requires in addition other factors such as low CD1d levels.

#### Infused Regulatory Cells Switch NKT-1 to a Regulatory Phenotype

**Figure 9:**
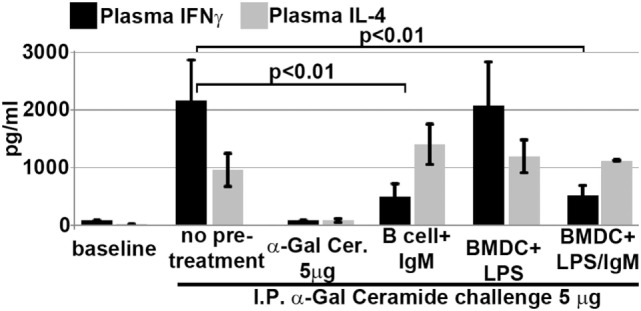
Infusion of IgM + LPS-pretreated BMDC and IgM-pretreated B cells induces *in vivo* NKT-1 cells to produce less IFNγ but normal or increased IL4 on α-gal-cer challenge. Intraperitoneal (IP) α-Gal-cer (5 μg) was given 24 h after cell infusion, and postchallenge plasma was obtained at 2 h for IL4 and at 18 h for IFN-γ. Mice initially treated with a 5-μg bolus (IP) of α-gal-cer produced very low IFNγ and IL4 upon re-challenge with α-gal-cer. Data are a mean of three separate experiments.

We next determined if intravenously infused regulatory BMDC and B cells protected mice from renal IRI by rendering *in vivo* NKT-1 non-functional or switching these cells to a regulatory phenotype. Twenty-four hours after infusion of *ex vivo* pretreated BMDC or B cells, the *in vivo* function of NKT-1 cells was tested by specifically activating NKT-1 cells with intraperitoneal α-gal-ceramide (5 μg) injection. Under these *in vivo* activating conditions, only NKT-1 cells are directly activated by α-gal-ceramide to secrete IL4 and IFN-γ, and release of these cytokines can be quantitated in plasma (23). Data in Figure 9 show that NKT-1 cells from mice infused with regulatory BMDC or pan-B cells are switched to a regulatory phenotype, as these NKT-1 cells produce significantly reduced levels of IFNγ but not IL4 when activated with α-gal-ceramide. NKT-1 cells were not rendered anergic as such anergic NKT-1 cells produce extremely low levels of both IFNγ and IL4 when activated with α-gal-ceramide (23). The latter is depicted in Figure 9 where *in vivo* NKT-1 cells were rendered anergic with a large bolus of α-gal-ceramide and then re-challenged 5 days later with α-gal-ceramide.

Data in Figures 7A and 8A and our prior studies (4) would indicate that infused regulatory pan-B cells and BMDC require lipid antigen presentation in the context of low CD1d expression, costimulatory interaction, as well as PD1/PDL1 and IL10 to switch *in vivo* NKT-1 to a regulatory phenotype. However, IgM induces regulatory B cells by a mechanism not involving IL10. *In vivo* NKT-1 cells with a regulatory phenotype fail to produce high levels of IFNγ (and induce an inflammatory response) when re-challenged with lipid antigens released after ischemic kidney injury.

### Low CD1d Expression on *Ex Vivo* Pretreated Cells Is Not Sufficient to Switch Them to a Regulatory Phenotype

Figure 5 also shows that some non-protective infused cells had downregulation of CD1d expression, thus indicating that low CD1d expression on B cells or BMDC by itself may be insufficient to regulate *in vivo* NKT-1 as the cross-talk between infused cells and *in vivo* NKT-1 is also influenced by levels of activating and inhibitory cytokines as well as costimulatory receptors that induce activating or inhibitory signaling pathways. For example, it is possible that the CD1d low LPS + IgM-pretreated pan-B cells did not regulate *in vivo* NKT-1 as these pretreated B cells expressed high levels of CD86 (Figure 5) and possibly produced high levels of pro-inflammatory cytokines.

### Infused Regulatory Cells Do Not Inhibit Ischemia-Induced Innate Inflammation by Regulating *In Vivo* DC or Innate Effectors or Enhancing Splenic Anti-inflammatory Cells

**Figure 10:**
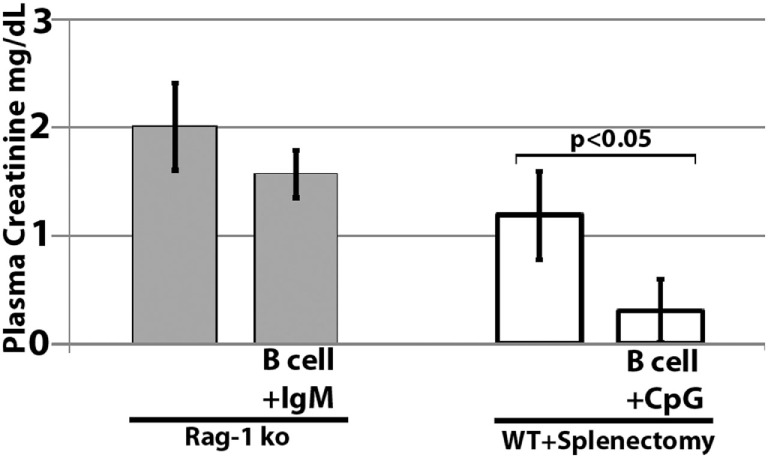
Infused regulatory B cells do not protect Rag-1 ko mice from ischemic acute kidney injury but protect mice that had prior splenectomy. *Ex vivo* pretreated regulatory B cells were infused in mice splenectomized 7 days before B cell infusion. Four mice were used in each group.

Further studies were performed in RAG-1 ko mice to determine if the infused IgM-pretreated B cells could ameliorate ischemia-induced inflammation by regulating *in vivo* DC or innate effectors (e.g., NK or granulocytes) *via* a mechanism not involving CD1d. To test this question, we infused IgM-pretreated pan-B cells into RAG-1 ko mice. As depicted in Figure 10, IgM-pretreated pan-B cells failed to protect RAG-1 ko mice from renal ischemia-induced renal injury, thus indicating that the infused regulatory B cells cannot directly regulate *in vivo* DC or innate effectors. In prior studies, we showed that infused IgM + LPS-pretreated regulatory BMDC also failed to protect RAG-1 ko mice from renal ischemia induced injury (4).

We next determined if there was an increase in splenic CD4^+^CD25^+^Foxp3^+^ T cells and CD4^+^CD25^+^IL10^+^ Treg cells when compared to mice with severe kidney injury. There was no difference in the level of these Treg cells between the two groups (data not shown) when splenic cells from protected mice were examined 24 h after renal ischemia, thus indicating that, in our renal ischemia model, protection was not mediated by an increase in splenic Tregs. However, we did not determine if there was an increase in Tregs several days after infusion of regulatory B cells or BMDC.

In murine models of ischemic AKI, prior splenectomy has been shown to worsen AKI (27) as well as AKI-induced lung injury (28). Potential mechanisms include removal of IL10 producing anti-inflammatory splenocytes as well as removal of anti-inflammatory splenocytes that are dependent on cholinergic stimulation in the spleen (27, 28). In addition, prior splenectomy has been shown to negate the protective effect of regulatory BMDC (i.e., S1P3 deficient), which, when infused, interact in the spleen with CD169^+^ marginal zone macrophages that induce Foxp3^+^ Tregs (29). Since significant numbers of infused cells also interact with splenocytes (21), we performed studies on mice splenectomized on day-7, to examine if infused regulatory B cells mediated protection by interacting with anti-inflammatory splenocytes. Data in Figure 10 clearly show that infused regulatory B cells do not require anti-inflammatory splenocytes to mediate protection. Instead, these data on prior splenectomized mice support the concept that infused regulatory cells, induced *ex vivo* by IgM or CpG, mediate protection by directly interacting and regulating *in vivo* NKT-1 cells that are also present in other organs, e.g., liver and bone marrow.

## Discussion

The current studies were initiated to determine the mechanism by which polyclonal natural IgM inhibits the inflammatory response following renal ischemia. We hypothesized that one potential mechanism involved binding of IgM to receptors on B cells and DCs especially since we noted several fold higher IgM binding to splenic B cells and DC when compared to T cells. To test our hypothesis, we pretreated BMDC or pan B cells with polyclonal IgM and demonstrated that these *ex vivo* pretreated cells, when intravenously injected 24 h before renal ischemia, protected WT-B6 mice from developing AKI, by inhibiting the innate inflammatory response to DAMPS released by ischemic tubules (4). In this kidney ischemia model, activation of NKT-1 amplifies the inflammatory response which is largely responsible for causing AKI (16). Several observations were made. First, we show that BMDC, pretreated *ex vivo* with IgM + LPS, or pan-B cells, pretreated *ex vivo* with IgM or CpG, can be switched to a “regulatory” phenotype with CD1d downregulation. Bregs and regulatory BMDC require CD1d-mediated interaction with NKT-1 to inhibit the innate inflammatory response as CD1d ko B cells or BMDC fail to function as Bregs (Figure 7A). Such *ex vivo* induced regulatory BMDC or pan-B cells, by presenting lipid antigen in the context of low CD1d, regulate *in vivo* NKT-1 cells, which when challenged with α-gal-ceramide produce normal or increased IL4 but low IFN-γ (Figure 9). However, our observations indicate that CD1d downregulation by itself is not sufficient for *ex vivo* induced regulatory pan-B cells or BMDC to switch *in vivo* NKT-1 to a regulatory phenotype. This is best exemplified by LPS + IgM-pretreated pan-B cells, which failed to regulate *in vivo* NKT-1 and protect mice from ischemia-induced AKI despite downregulation of CD1d (Figures 1 and 5). These observations indicate that other mechanisms such as inhibitory costimulatory receptors and the balance between pro-inflammatory and anti-inflammatory cytokines are also involved in switching pan-B cells to regulatory cells (Figure 8B). Second, we show that CpG-induced regulatory B cells differ from IgM-induced regulatory B cells. Regulatory B cells induced by CpG require PDL1 (but not PD1) and IL10 to inhibit *in vivo* NKT-1 cells (Figure 8A) (16), while IgM-induced regulatory B cells require PDL1 but do not require IL10 or TGFβ1 to regulate *in vivo* NKT-1 (Figure 8A). It is possible that IgM-pretreated pan-B cells, which lack pro-inflammatory cytokines (Figure 8B), regulate *in vivo* NKT-1 cells by utilizing only PDL1 or in addition by producing other inhibitory molecules such as adenosine or Tim-1 as has been shown with certain other Bregs [reviewed in Ref. (30)]. In the previous study, we show that BMDCs, unlike B cells, require PD1 (but not PDL1) and IL10 to mediate their *in vivo* regulatory activity (4). Finally, we show that infused cells regulate *in vivo* NKT-1 cells within 24 h, and this may explain why IgM-pretreated human B cells induced regulatory activity in mice (Figure 4) even though they are most likely rejected within 3 to 4 days after being infused.

The current data and data from our prior studies (4) indicate that the cross-talk between inhibitory signals induced by IgM and signaling induced by TLRs 4 and 9 agonists have different functional effects on B cells and BMDC. First, LPS negates the IgM-induced regulatory activity in B cells while enhancing the IgM-induced regulatory activity in BMDC (Figure 1), and second, CpG induces regulatory activity in B cells but not in BMDC (Figure 1). A potential explanation for these differing effects is that LPS and CpG may bind to other receptors (in addition to TLR) on B cells and BMDC. For example, there is evidence to show that CpG, by binding to the B1 class of scavenger receptors on B cells, regulates B cells (31). This may have clinical relevance as the presence of excess TLR9 ligands released by pathogens or apoptotic cells could induce regulatory activity in B cells and inhibit inflammation mediated by NKT-1.

Currently, studies are in progress to determine how IgM regulates B cells and BMDC. It is possible that IgM binds to different inhibitory receptors on B cells and BMDC and, as a result, activates different inhibitory signaling pathways that differentially affect the cross-talk with pathways induced by TLRs 4 and 9 ligands. IgM can regulate B cells by activating inhibitory receptors that normally inhibit B cell activation. B cells express a number of “inhibitory” receptors namely FcγRIIB, CD22, siglec G, PD1, PDL1, CD5, and CD72. This entire family of “inhibitory” receptors recruits SHP-1 phosphatases to inhibit NFκB activation induced by BcR or TLR activation (32). Siglec receptors (i.e., CD22 and siglec G) are likely candidates as they are expressed on all B cells and IgM binds to these receptors (33, 34). Regulating B cells by IgM binding to FcμR is also a likely possibility as FcμR is expressed on all splenic B cells (35), and there are data to show that FcμR regulates B cell apoptosis, immunoglobulin production (including natural IgM) [reviewed in Ref. (36, 37)], and the density of membrane bound IgM on the BcR receptor (37). In addition, it is possible that B cells, like DC (38), can be regulated by internalization of FcμR bound to natural IgM/autoantigen complexes.

Our IgM-induced regulatory pan-B cells differ from previously described regulatory B cell subsets that regulate autoimmune-mediated inflammation (30) in that (i) they express low levels of CD1d (Figure 5A), (ii) their regulatory activity is negated by TLR4 activation (Figures 1 and 2), (iii) they inhibit innate inflammation by regulating *in vivo* NKT-1 cells, and (iv) they do not require IL10 for their regulatory activity (Figure 8A). Furthermore, we show that IgM can induce regulatory activity in B cell subsets arising from different splenic compartments, i.e., CD5^+^ B1 cells, FO B2 cells, and pan-B cells (Figure 3B). In our studies, we did not attempt to evaluate the regulatory effect of infused B cells on *in vivo* splenic Tregs as mice were sacrificed after 48 h of infusion. Currently, it is unclear how CpG induces regulatory activity in murine splenic pan-B cells but not in BMDC, and second, how LPS negates IgM-induced regulatory activity in B cells but not in BMDC, even though both TLR agonists increase IL10 production. We have not defined the mechanism by which IgM or CpG downregulates CD1d or induces regulatory activity in murine splenic pan-B cell subsets.

We have shown in prior *in vitro* studies that IgM-ALA (i) inhibits human and murine T cell activation and proliferation in response to anti-CD3 or alloantigens by binding and downregulating CD4 and also by binding to CD3 and inhibiting Zap70 phosphorylation in response to anti-CD3, (ii) inhibits differentiation of naïve murine T cells into TH1 and TH17 effectors, (iii) enhances generation of human and murine Foxp3^+^ T regs, and (iv) binds to human CCR5 and CXCR4 and inhibits leukocyte chemotaxis in response to chemokines (3, 9). The current studies provide another potential mechanism for IgM-ALA to regulate inflammation, i.e., reprogramming DC and pan-B cells to switch to a CD1d^lo^ regulatory phenotype, which in turn regulates NKT-1 cells.

We propose the following hypothesis based on our findings. Ischemic injury or infectious agents activate DC and B cells with upregulation of costimulatory molecules, cytokine production, and antigen-presenting receptors to induce an inflammatory response. B1 cells are also activated *via* BCR and TLR, especially with infectious agents, and rapidly increase the production of natural IgM, which binds to certain undefined receptors on DC and B cells and switches these cells to a regulatory phenotype. TLR9 agonists, released by pathogens or apoptotic cells, could also provide another mechanism to regulate pan-B cells in the event of excess LPS or other TLR agonists. IgM-induced regulatory DC and B cells require PD1/PDL1, IL10, and possibly other mediators to mediate suppression and together with other *in vivo* regulatory mechanisms (e.g., Bregs, Tregs, IL10, FcγRIIB, and IgM) regulate the inflammatory process.

## Ethics Statement

All experiments were performed in accordance with the recommendations of the National Institutes of Health and Institutional Animal Care and Use Guidelines. The Animal Research Committee of the University of Virginia approved all procedures and protocols.

## Author Contributions

PL conceived the idea, designed the experiments, analyzed data, and wrote the manuscript. MO supervised the kidney ischemia experiments and critically reviewed the data and manuscript. KS performed experiments and prepared the figures. LH performed kidney ischemia experiments and AB performed kidney histology.

## Conflict of Interest Statement

The authors declare that the research was conducted in the absence of any commercial or financial relationships that could be construed as a potential conflict of interest.
